# Reply to Comments: Comparison of Methods Study between a Photonic Crystal Biosensor and Certified ELISA to Measure Biomarkers of Iron Deficiency in Chronic Kidney Disease Patients

**DOI:** 10.3390/s20041149

**Published:** 2020-02-19

**Authors:** Ross D. Peterson, Kenneth R. Wilund, Brian T. Cunningham, Juan E. Andrade

**Affiliations:** 1 Department of Food Science and Human Nutrition, University of Illinois at Urbana-Champaign, Urbana, IL 61801, USA; rdpeter2@illinois.edu; 2 Department of Kinesiology and Community Health, University of Illinois at Urbana-Champaign, Urbana, IL 61801, USA; kwilund@illinois.edu; 3 Division of Nutritional Sciences, University of Illinois at Urbana-Champaign, Urbana, IL 61801, USA; 4 Department of Electrical and Computer Engineering, University of Illinois at Urbana-Champaign, Urbana, IL 61801, USA; bcunning@illinois.edu; 5 Department of Bioengineering, University of Illinois at Urbana-Champaign, Urbana, IL 61801, USA

In this brief note, we respond to the comments made by Dr. N. Abbas, who raised a concern over the display of information in a graph presented in our article “Comparison of Methods Study between a Photonic Crystal Biosensor and Certified ELISA to Measure Biomarkers of Iron Deficiency in Chronic Kidney Disease Patients” (Peterson, et al. 2017, 17(10), 2203). We have carefully analyzed the arguments in the comment and have concluded that in effect there is an error in the display of the estimated error boundaries in Figure 2. These boundaries have been corrected and are shown in the new Figure 2 presented below. This was an error at the final stages of rendering this figure, in which the boundaries for the 0 ± 1σ(δ) and 0 ± 2σ(δ) were moved. This error in the display of boundaries does not change the discussion of findings or the conclusions.

## Figures and Tables

**Figure 2:**
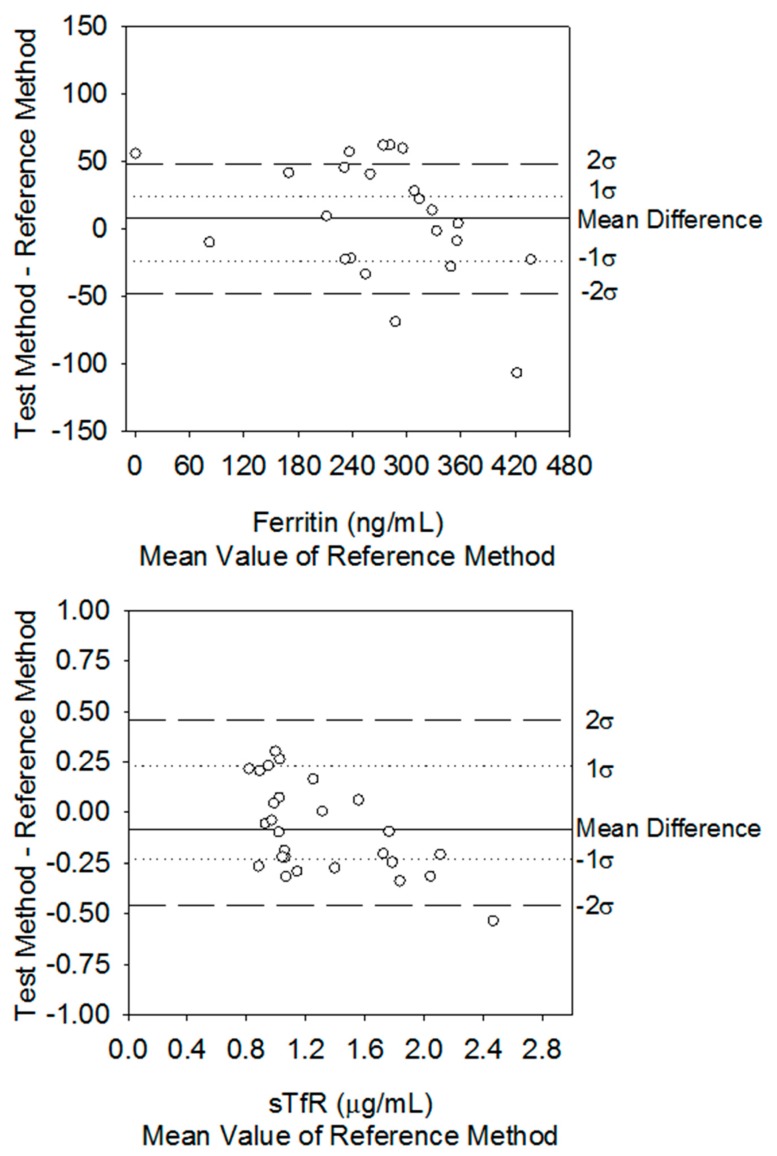
Difference plots comparing serum ferritin and sTfR concentrations from hemodialysis patients using the PC biosensor against the certified ELISAs.

